# Analysis of second grade hybrid nanofluid flow over a stretching flat plate in the presence of activation energy

**DOI:** 10.1038/s41598-022-22460-1

**Published:** 2022-12-13

**Authors:** Muhammad Arif, Anwar Saeed, Panawan Suttiarporn, Waris Khan, Poom Kumam, Wiboonsak Watthayu

**Affiliations:** 1 Fixed Point Research Laboratory, Fixed Point Theory and Applications Research Group, Center of Excellence in Theoretical and Computational Science (TaCS-CoE), Faculty of Science, King Mongkut’s University of Technology Thonburi (KMUTT), 126 Pracha Uthit Rd., Bang Mod, Thung Khru, Bangkok, 10140 Thailand; 2 Center of Excellence in Theoretical and Computational Science (TaCS-CoE), Faculty of Science, King Mongkut’s University of Technology Thonburi (KMUTT), 126 Pracha Uthit Rd., Bang Mod, Thung Khru, Bangkok, 10140 Thailand; 3 Faculty of Science, Energy and Environment, King Mongkut’s University of Technology North Bangkok, Rayong Campus, Rayong, 21120 Thailand; 4 Department of Mathematics and Statistics, Hazara University Mansehra, Khyber Pakhtunkhwa, 21120 Pakistan; 5 Department of Medical Research, China Medical University Hospital, China Medical University, Taichung, 40402 Taiwan

**Keywords:** Engineering, Mathematics and computing

## Abstract

The research of fluid containing nanoparticles for the heat transport characteristics is very famous because of its variety of real-life applications in various thermal systems. Although the thermal efficiency of the nanofluid was effective but still the nano scientists were trying to introduce some new advance class of fluid. Therefore, an advance class of fluid is developed by the dispersion of two different nano sized particles in the conventional base fluid known as “Hybrid nanofluid” which is more effective compared to simple nanofluids in many engineering and industrial applications. Therefore, motivated from the hybrid type of nanofluids in the current research we have taken two-dimensional laminar and steady flow of second grade fluid passing through porous plate. The engine oil base fluid is widely used fluid in the engineering and industrial problems. Keeping these applications in mind the engine oil is considered and two different nanoparticles Copper and aluminum oxide are added in ordered to get the required thermal characteristics. In addition to this the thermal radiation, chemical reaction, activation energy, Brownian motion and thermophoresis are also addressed during the current research. The present proposed higher-order PDE’s is transformed to the non-linear system of ODE’s. For the solution of the proposed high non-linear model HAM method is employed. As the hybrid nanofluid are highlighted on the second-grade fluid flow over a horizontal porous flat plate. During the present analysis and experimental study, it has been proved that the performance of hybrid nanofluid is efficient in many situations compared to nanofluid and regular fluid. For physical interpretation all the flow parameters are discussed through graphs. The impact of volume fraction is also addressed through graphs. Moreover, the comparative analysis between hybrid and nanofluid is carried out and found that hybrid nanofluid performed well as compared to nanofluid and regular fluid. The engineering quantities obtained from the present research have been presented in tables.

## Introduction

Engine oil is widely used fluid for many engineering problems and has enormous applications in automobile, heat engine, cooling systems, transformer etc. The regular engine oil have low thermal properties in improve these properties initially, it was proved by Choi^1^, that when nanomaterials are added in a conventional fluid will improve heat transfer properties of the working fluids with good stability. Afterwards, many researchers take interest in the research of nanofluid. Nanofluids have useful applications in different cooling systems, engineering, biology, medicine, nuclear power systems, heat generators, heat turbines, automobile engine and agriculture. Shahzad et al.^2^ explored a research by considering engine oil and found the efficient increase in the base fluid engine oil by taking molybdenum disulfide nanoparticles. Arif et al.^3^ where the authors inspected thermal transport features of different particles in engine oil and calculated that thermal characteristics can be improved by the addition of nanoparticles in the working fluid engine oil in different operating systems. Sepyani et al.^4^ examined an experiment and found that addition of ZnO nanoparticles in engine oil improve the thermal transport properties as well as lubricity. Patil et al.^5^ reported a review on collecting a data by suspending different nanoparticles in engine oil base fluid for long life span and increase the lubricity and working capability of engine oil. Krishna et al.^6^ investigated the impact of radiation on the flow of nanofluid for various thermal applications in different industrial problems. Similarly, Puneeth et al.^7^ explored the theoretical studies by highlighting the thermal features using the applications of nanofluid with the use of stretching sheet in many physical problems. In another paper Krishna and Chamkha^8^] developed nanofluid flow model and its applications using rotating boundary layer flow passes through vertical plate with various advance applications.

Although the performance of nanofluid in different operating systems was good and shows good thermal performance. But still the researchers need to advance cooling properties which cannot be obtained from the simple nanofluid. Recently, the idea developed by adding two particles forming mixture in a base fluid was studied and found the promising performance in the resultant fluid. When the composition of two or more nanoparticles added and mixed in base fluid then the obtained fluid is known as hybrid nanofluid become more efficient fluid in various circumstances. Recently, many researchers took interest in the research of hybrid nanofluid and use in different engineering and medical sciences, like heat exchangers, heat pumps, heat turbines, cooling of electrical circuits, etc. The study of engine oil-based hybrid nanofluid investigated by Asadi et al.^9^, inspected engine oil performan ce by dissolving the mixture of MWCNT and Zno nanoparticles for improving the heat properties and lubrication performance. Sulgani et al.^10^ explained the improvement in the thermal transport efficiency of engine oil by taking the various temperature scales and adding the composite of Al_2_O_3_/Fe_2_O_3_ nanoparticles. In similar manner, Arif et al.^11^ explored in their studies the temperature enhances due to the dispersion of GO-MoS_2_ nanoparticles in engine oil making hybrid nanofluid and the fluid is assume to flow over an oscillating cylinder. Jamshed et al.^12^ discussed the entropy analysis using the mixture of nanoparticles in engine oil for advance applications. Krishna et al.^13^ carried a research by highlighting the flow of Casson fluid passes through exponentially accelerated surface with the fluid having two nanoparticles forming hybrid nanofluid using the applications in various cooling systems. Shah et al.^14^ where the authors inspected the hybrid nanofluid and its physical applications by highlighting the chemical reaction influence and motile microorganism using stretching sheet applications. Similarly, Kavya et al.^15^ investigated magnetic hybrid nanofluid and its useful applications in a variety of physical situations. In addition to this the authors studied the thermal features of MoS_2_ and copper hybrid nano composite fluid flow over stretching cylinder. Furthermore, Ghalambaz et al.^16^ and Mehryan et al.^17^ inspected some uniform thermal features of the hybrid nanofluid in a square cavity by highlighting various practical thermal applications in different circumstances. In another paper, Mehryan et al.^18^ where the authors decided to inspect the numerical study of the fluid containing the double nanoparticles at the same time for advance cooling applications and the fluid is assumed to flow inside a porous encloser.

The nature have two major classes of fluids i.e., Newtonian and non-Newtonian. Some practical situations where Newtonian fluid is used but many real-world problems exists which can’t be explained by the simple fluid. Therefore, the complex nature of the fluid can be highlighted by employing the non-Newtonian fluid models. In order to highlight these complex features of real phenomena non-Newtonian fluids play a vital rule in the investigation of such complex fluid flow problems. The non-Newtonian fluid have a attracted the attention because of novel applications in industries and engineering problems. The non-Newtonian fluids commonly applied in the food processing, polymeric material and have various applications in chemical processes petroleum engineering problems. The example of such fluids is paint, ketchup, honey, blood, starch, tooth paste, engine oil, etc. among the non-Newtonian fluid Second grade is famous fluid which have considerable attention in various physical situations. Motivated from the complex features of non-Newtonian fluids, Jawad et al.^19^ inspected the dynamical behavior of second grade hybrid nanofluid with the radiation subject to Lorentz force. To describe the dynamics of such fluids, Roy and Pop^20^ examined the flow analysis of a second-grade hybrid nanofluid passes through stretching/shrinking sheet. Imran et al.^21^ developed the computational analysis of nanoparticles shaped on second grade hybrid nanofluid flow using the advance applications of solar collector. Alzahrani et al.^22^ explored the flow analysis of second hybrid nanofluid over a flat plate due to solar radiation. Mamaloukas et al.^23^ demonstrated the impact of second grade fluid with applications in various engineering problems. In addition to this motivated from the applications of non-Newtonian fluid recently Modather et al.^24^, Krishna et al.^25^ and VeeraKrishna^26^ explored some unique features of non-Newtonian fluid in different physical situations. Some other related studies of non-Newtonian fluids and its unique features can be found in^27–31^.

The impact of thermal radiations has enormous applications in different fluid flow problems. The practical applications of thermal radiation are highlighted using the micropolar fluid passes through stretching sheet was investigated by Naveed et al.^32^. During the analysis they explains different applications of thermal radiation in physical problems. Similarly, Ali et al.^33^ investigated the impact of thermal radiation on the fluid flow profile with application of chemical reaction and non-uniform temperature at the boundary. Wang Ali et al.^34^ discussed the presence of thermal radiation phenomena and explained some useful features of the radiation in the dynamics of various fluid flow problems. Navarro et al.^35^ studied thermal radiation experimentally and explained the extreme kinds of unsteady flow (turbulent) in the presence of radiation using cavity fluid flow problems. Siegel et al.^36^ explored thermal effect in various physical applications like the authors calculated radiation and its applications in the engines turbine and discussed that turbine vanes and blades are important for temperature reduction in various situations. Bataller^37^ discusses some advance applications of thermal radiation effect in the Blasius type flow. In addition to this motivated from the advance applications of thermal radiation in different physical situation Chamkha^38–41^ inspected some real-life applications in various physical situations.

The phenomena of fluid flow on the stretching surfaces have unique industrial and engineering supplications especially, in electrochemistry and manufacturing of polymers. Stretching sheet applications in the flow dynamics can be used for the preparation of glass and paper. Cortell^42^ investigated the radiative fluid flow with the involvement of stretch surface and thermal effects. Gowda et al.^43^ examined in their research that the ferromagnetic nanofluid is allowed to pass through stretched surface with practical applications in the presence of magnetic dipole effect. Ziabakhsh et al.^44^ where the authors tried to develop an analytical solutions diffusion type flow with chemical effect and the fluid is allowed to pass through stretch sheet for advance engineering and industrial applications. Hayat et al.^45^ inspected the flow of second grade fluid using the stretching sheet surface with a detail explanation of a variety of daily life uses which include heat and mass transport phenomena. Dessie and Kishan^46^ carried research explaining the stretching sheet applications by highlighting the MHD effect in the presence of heat source variable viscosity and viscous dissipation. Layek et al.^47^ and Bhargava et al.^48^ considered the fluid flow through stretching porous surface using various physical phenomena in the presence of heat production effect and explain various daily life applications. The increasing interest of recent research shows that stretching sheet has a variety of applications in modern science and engineering problems. Motivated from these novel applications recently, Sreedevi et al.^49^ and Chamkha^50^ where the authors investigated some useful applications of stretching sheet phenomena and explain various physical situations where stretching sheet is applied.

In the last few decades, the research of activation energy and its applications in a variety of modern sciences like chemical engineering biological processes, fuels technology, and pyrolysis process has increased significantly. These novel applications become the main motivation of the researchers and scholars which are attracted to consider the impact of activation energy in various physical situations. Keeping these unique applications in mind recently, Cai et al.^51^ examined a thorough review by considering the presence of activation energy model and discussed its applications in the pyrolysis of lignocellulosic biomass. In next research paper, Cai and Liu^52^ use the same model for activation energy and focused to highlight some advance and novel applications of such energy models. Moreover, some polymeric properties of the activation energy were highlighted by Li et al.^53^ where the authors mentioned polymer pyrolysis properties of activation energy model. Further they discussed the activation energy mode selection, characteristics, validation of the model and its sensitive analysis. Henda et al.^54^ elevated the advance applications bioconvection assessment of the magnetized third grade nanofluid along with the applications of thermal radiation and exponential effect.

The research of computational fluid dynamics has widely used and become a versatile and powerful tool for investigating the chemical engineering problems. In modern era, many mathematical models have been used for chemical processes and its novel real-life applications in different circumstances. Raman et al.^55^ explored the chemical processes and its uses on the computational fluid dynamics in various physical situations. Similarly, the chemical processes was highlighted by Kuipers et al.^56^ by considering the mathematical modeling in computational fluid dynamics for evaluating different chemical processes. Lian et al.^57^ anticipated the impact of chemical processes and chemical reaction kinetics and Ozonation processes. Micale et al.^58^ scrutinized computational fluid dynamics CFD with chemically reacting fluid flows at surfaces for many physical applications. Keil et al.^59^ deliberated a scientific investigation computing in chemical engineering and its application in different physical situations using the computational fluid dynamics. Magyari and Chamkha^60^ explored a variety of industrial and engineering applications of chemical reactions using stretch surface.

The present research work evaluated numerical analysis of incompressible, laminar steady second grade hybrid nanofluid flow over a horizontal stretching flat plate. To the best of our knowledge no work is reported to explain the thermal and concentration performance of second grade fluid model with the composition of aluminum oxide Al_2_O_3_ and copper Cu nanoparticles uniform dispersion in engine oil. The main motivation of mixing two particles at the same time is to calculate heat enhancement more effectively. Moreover, in the current study we also addressed the presence of radiation effect on the temperature profile, chemical process and activation energy is considered in the concentration of the fluid are also considered. The solutions of the present high order non-linear problem have been obtained by using the HAM method. All the flow parameters are highlighted through figures for explaining more physicals perspectives of the considered pertinent flow parameters. In addition, in the present analysis Al_2_O_3_ and Cu are mixed in engine oil for advance thermal characteristics. Finally, second grade fluid parameter is shown during the dynamics of the fluid through stretching sheet, the influence of volume friction is also addressed. The engineering quantities are calculated for mass, temperature and velocity equation and presented in tabular form.

## Mathematical modeling

**Figure 1:**
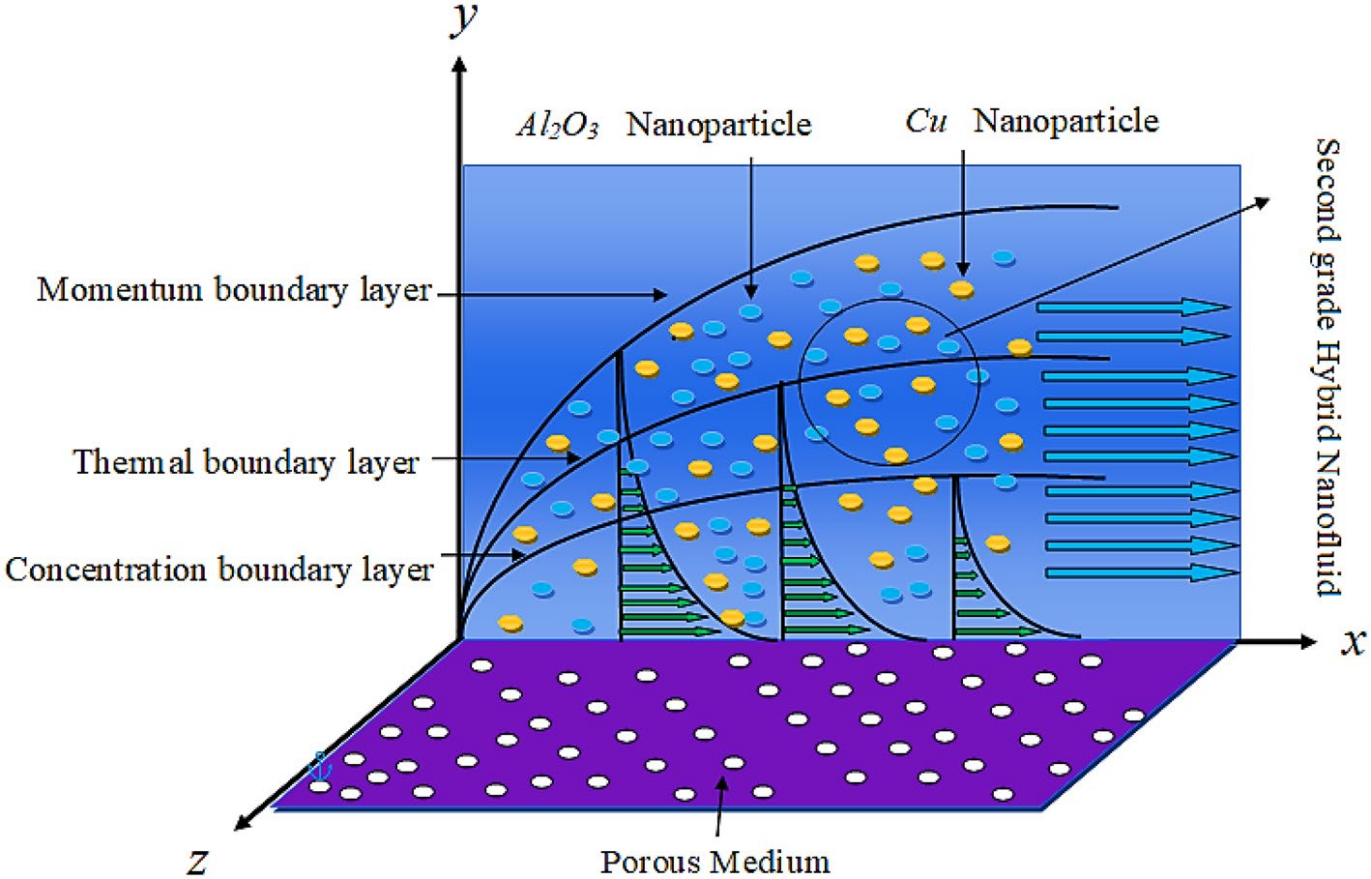
Geometry of the Problem.

The present analysis addressed non-Newtonian second grade fluid laminar flow through stretching porous surface. The second-grade fluid is assumed as laminar, steady, and incompressible two-dimensional flow. In the current research engine oil is used as base fluid by adding two kinds of nanoparticles to form a hybrid. Hybrid fluid is a new advance class of fluid have better thermal properties compared to nanofluid. Furthermore, the impact of radiative heat effect, viscous dissipation, activation energy and chemical reaction are also considered in the present analysis. The physical sketch is provided in Fig. 1.

## Formulation of the flow analysis

The current research elevate second grade fluid using stretching sheet applications under the irregular extending rate which can be expressed as:

1 $$ U_{w} \left( {x,t} \right) = bx, $$

where b shows stretching porous sheet. The temperature at the flat surface can be expressed as $T_{w} \left( {x,t} \right) = T_{\infty } + b^{*} x,$ here it is assumed that when $x = 0,$ we have $b^{*} ,\,\,T_{w} \,{\text{and}}\,T_{\infty }$, represents the rate of temperature difference, wall and ambient temperature respectively. A homogenous incompressible second grade fluid has a constitutive equation presented in^26^, the stress tensor can be expressed as follows:

2 $$ {\varvec{T}} = \mu {\varvec{A}}_{m1} + \alpha_{1} {\varvec{A}}_{m2} + \alpha_{2} {\varvec{A}}_{m1}^{2} - pI, $$

Here ${\varvec{T}}$ and $p$ represents Cauchy Stress and pressure, $\mu$, $\alpha_{1} \,\,{\text{and}}\,\,\alpha_{2}$ shows the is the dynamic viscosity and material variables respectively, while the kinematic tensors here represented by ${\varvec{A}}_{m1} \,{\text{and}}\,{\varvec{A}}_{m2}$ and it can be expressed as:

3 $$ {\varvec{A}}_{m1} \, = \left( {gradV} \right){ + }\left( {gradV} \right)^{T} , $$

4 $$ {\text{and}}\; \, {\varvec{A}}_{m2} = \frac{{\partial {\varvec{A}}_{m1} }}{\partial t} + {\varvec{A}}_{m1} \left( {gradV} \right){ + }{\varvec{A}}_{m1} \left( {gradV} \right)^{T} , $$

where $\frac{\partial }{\partial t}$, express the time dependent derivative, $V$ shows the velocity field of the flow, here it is considered that the flow is incompressible therefore, Clasius-Duhem inequality (i.e., $\mu \ge 0,\,\alpha_{1} \ge 0\,\,{\text{and}}\,\,\alpha_{1} + \alpha_{2} = 0$) and $\nabla = \left( {\frac{\partial }{\partial x},\,\frac{\partial }{\partial y},\,\frac{\partial }{\partial z}} \right)$.

The continuity equation for such flow can be expressed a:

5 $$ Div(V) = 0, $$

and

6 $$ \rho \left[ {\frac{\partial V}{{\partial t}} + \left( {V.\nabla } \right)V} \right] = Div\left( T \right). $$

In the present study we assume two-dimensional steady and laminar flow, therefore velocity velocity can be expressed as follows:

7 $$ V = \left( {u\left( {x,y} \right),v\left( {x,y} \right),0} \right), $$

## Governing equations and transformation analysis

In the present study we have considered two-dimensional second grade hybrid nanofluid flow over a porous horizontal flat plate. The continuity equation to be satisfied given as under:

8 $$ \frac{\partial u}{{\partial x}} + \frac{\partial v}{{\partial y}} = 0, $$

The momentum equation in component form can be expressed as:

9 $$  \begin{aligned}   u\frac{{\partial u}}{{\partial x}} + v\frac{{\partial u}}{{\partial y}} =  & U_{\infty } \frac{{\partial U_{\infty } }}{{\partial x}} + \frac{{\alpha _{1} }}{{\rho _{{hnf}} }}\left[ {\frac{{\partial u}}{{\partial x}}\left( {\frac{{\partial ^{2} u}}{{\partial y^{2} }}} \right) + u\left( {\frac{{\partial ^{3} u}}{{\partial x\partial y^{2} }}} \right) + \frac{{\partial u}}{{\partial y}}\left( {\frac{{\partial ^{2} v}}{{\partial y^{2} }}} \right) + v\left( {\frac{{\partial ^{3} u}}{{\partial y^{3} }}} \right)} \right] \\     &  + \frac{{\mu _{{hnf}} }}{{\rho _{{hnf}} }}\left( {\frac{{\partial ^{2} u}}{{\partial y^{2} }}} \right) - \frac{{\mu _{{hnf}} }}{{\rho _{{hnf}} .k}}u, \\  \end{aligned}   $$

The heat equation of the present flow in component form is given as:

10 $$ \begin{aligned}   u\frac{{\partial T}}{{\partial x}} + v\frac{{\partial T}}{{\partial y}} =  & \frac{{k_{{hnf}} }}{{\left( {\rho C_{p} } \right)_{{hnf}} }}\left( {\frac{{\partial ^{2} T}}{{\partial y^{2} }}} \right) - \frac{1}{{\left( {\rho C_{p} } \right)_{{hnf}} }}\left( {\frac{{\partial q_{r} }}{{\partial y}}} \right) \\     &  + \frac{{\mu _{{hnf}} }}{{\left( {\rho C_{p} } \right)_{{hnf}} }}\left( {\frac{{\partial u}}{{\partial y}}} \right)^{2} \frac{{\alpha _{1} }}{{\left( {\rho C_{p} } \right)_{{hnf}} }}\left[ {u\frac{{\partial u}}{{\partial y}}\frac{{\partial ^{2} u}}{{\partial x\partial y}} + v\left( {\frac{{\partial u}}{{\partial y}}} \right)\left( {\frac{{\partial ^{2} u}}{{\partial y^{2} }}} \right)} \right]. \\  \end{aligned}   $$

Finally, the concentration in the component form is given as:

11 $$ u\frac{\partial C}{{\partial x}} + v\frac{\partial C}{{\partial y}} = \left( {D_{B} } \right)_{hnf} \left( {\frac{{\partial^{2} C}}{{\partial y^{2} }}} \right) - \frac{{D_{T} }}{{T_{\infty } }}\left( {\frac{{\partial^{2} C}}{{\partial y^{2} }}} \right) - K_{1} r^{2} \left( {C - C_{\infty } } \right)\left( {\frac{T}{{T_{\infty } }}} \right)^{2} \exp \left( {\frac{{ - E_{a} }}{kT}} \right), $$

Here $u\,\,{\text{and}}\,\,v\,$ components of velocity, $U$ shows free stream velocity, $T\,\,{\text{and}}\,\,C$ are the temperature and concentration, $T_{\infty } \,\,{\text{and}}\,\,C_{\infty }$ are the ambient temperature and concentration respectively, $\alpha_{1}$ the parameter of second grade fluid, $k$ shows porosity, $q_{r}$ shows thermal radiation, $D_{B} \,\,{\text{and}}\,\,\,D_{T}$ are the Brownian and thermophoretic diffusion coefficient, $K_{1} r^{2}$ represents chemical reaction, $E_{a}$ shows activation energy, $\rho_{hnf}$ and $\mu_{hnf}$ represents the density and kinematic viscosity $k_{hnf}$ represents thermal conductivity, $C_{p}$ is the specific heat and $\left( {D_{B} } \right)_{hnf}$ represents mass diffusivity and the subscript hnf is used for hybrid nanofluids.

The radioactive heat flux for thermal radiation is given as:

12 $$ q_{r} = - \frac{{4\sigma^{*} }}{{3k^{*} }}T_{y}^{4} = - \frac{{16\sigma^{*} }}{{3k^{*} }}T^{3} T_{y} , $$

The BC’s to be satisfied are expressed as:

13 $$ \left. \begin{gathered} u\left( {x,o} \right) = U_{w} ,\,\,\,\,\,v\left( {x,0} \right) = V_{w} ,\,\,\,\,T = T_{w} ,\,\,C = C_{w} ,\,\,\,\,\,{\text{at}}\,\,{\text{y = 0}} \hfill \\ u \to U_{\infty } ,\,\,\,\frac{\partial u}{{\partial y}} \to 0,\,\,\,T \to T_{\infty } ,\,\,C \to C_{\infty } ,\,\,\,\,{\text{as}}\,\,y \to \infty . \hfill \\ \end{gathered} \right\}. $$

The nanofluid expressions for $\rho_{hnf} ,\mu_{hnf} ,\left( {\rho C_{p} } \right)_{hnf} ,k_{hnf}$ and $D_{hnf}$ are given by^11^:

14 $$ \begin{aligned} \rho_{hnf} = & \rho_{f} \left[ {\left( {1 - \phi_{hnf} } \right) + \frac{1}{{\rho_{f} }}\phi_{{Al_{2} O_{3} }} \rho_{{Al_{2} O_{3} }} + \frac{1}{{\rho_{f} }}\phi_{Cu} \rho_{Cu} } \right], \, \mu_{hnf} = \mu_{f} \left( {1 - \left( {\phi_{{Al_{2} O_{3} }} + \phi_{Cu} } \right)} \right)^{ - 2.5} , \\ \left( {\rho C_{p} } \right)_{hnf} = & \left( {\rho C_{p} } \right)_{f} \left[ {\left( {1 - \phi_{hnf} } \right) + \frac{1}{{\left( {\rho C_{p} } \right)_{f} }}\phi_{{Al_{2} O_{3} }} \left( {\rho C_{p} } \right)_{{Al_{2} O_{3} }} + \frac{1}{{\left( {\rho C_{p} } \right)_{f} }}\phi_{Cu} \left( {\rho C_{p} } \right)_{Cu} } \right], \\ k_{hnf} = & k_{f} \left[ {\frac{{2k_{f} + \frac{\left( A \right)}{{\phi_{hnf} }} + 2\left( A \right) - 2k_{f} \phi_{hnf} }}{{2k_{f} + \frac{\left( A \right)}{{\phi_{hnf} }} + \left( A \right) - k_{f} \phi_{hnf} }}} \right],\,\,\,\,\,{\text{where}}\,\,A = \phi_{{Al_{2} O_{3} }} k_{{Al_{2} O_{3} }} + \phi_{Cu} k_{Cu} \\ D_{hnf} = & \left( {1 - \left( {\phi_{{Al_{2} O_{3} }} + \phi_{Cu} } \right)} \right)D_{f} ,\,\, \\ \end{aligned} $$

The similarity transformation have been formed by suing the following variables:

15 $$ \left. {\xi \left( {x,y} \right) = \sqrt {\frac{b}{{\nu_{f} }}} y,\,\,\,\,\,u = bxf^{^{\prime}} \left( \xi \right),\,\,\,\,v = - \sqrt {\nu_{f} b} \,f\left( \xi \right),\,\,\,\,\,\theta \left( \xi \right) = \frac{{T - T_{\infty } }}{{T_{w} - T_{\infty } }},\,\,\,\Phi \left( \xi \right) = \frac{{C - C_{\infty } }}{{C_{w} - C_{\infty } }},\,\,} \right\}. $$

The transformed model can be expressed as:

16 $$ f^{\prime \prime \prime } \left( \xi \right) + \chi_{1} \chi_{2} \left[ \begin{gathered} \left( {f\left( \xi \right)f^{\prime \prime } \left( \xi \right) - \left( {f^{\prime}\left( \xi \right)} \right)^{2} } \right) + \hfill \\ \Gamma \left( {2f^{\prime}\left( \xi \right)f^{\prime \prime \prime } \left( \xi \right) - \left( {f^{\prime \prime } } \right)^{2} f\left( \xi \right) - f\left( \xi \right)f^{iv} \left( \xi \right)} \right) \hfill \\ \end{gathered} \right] - Kf^{\prime } \left( \xi \right) = 0, $$

17 $$ \chi_{4} \theta^{\prime \prime } \left( \xi \right)\left( {\chi_{4} + Nr} \right) + \chi_{3} \Pr \left( \begin{gathered} f\left( \xi \right)\theta^{\prime } \left( \xi \right) \hfill \\ + \frac{Ec}{{\chi_{1} }}\left[ {\left( {f^{\prime \prime } \left( \xi \right)} \right)^{2} + \Gamma \left( {f^{\prime } \left( \xi \right)\left( {f^{\prime \prime } \left( \xi \right)} \right)^{2} - f\left( \xi \right)f^{\prime \prime } \left( \xi \right)f^{\prime \prime \prime } \left( \xi \right)} \right)} \right] \hfill \\ \end{gathered} \right) = 0, $$

18 $$ \begin{aligned} \chi_{5} .\,\Phi^{\prime \prime } \left( \xi \right) + & \frac{Nt}{{Nb}}\theta^{\prime \prime } \left( \xi \right) + f\left( \xi \right)\Phi^{\prime } \left( \xi \right).Sc \\ & - K_{2} .Sc.\Phi \left[ {\sigma_{1} \theta \left( \xi \right) + 1} \right]\exp \left[ { - \frac{{E_{1} }}{{\sigma_{1} \theta \left( \xi \right) + 1}}} \right] = 0 \\ \end{aligned} $$

The physical BCs are to be satisfied:

19 $$ \left. \begin{gathered} f\left( 0 \right) = S,\,\,\,\,f^{\prime } \left( 0 \right) = 1,\,\,\,\,\theta \left( 0 \right) = 1,\,\,\,\Phi \left( 0 \right) = 1,\,\,\,\,\,\,{\text{at}}\,\,\xi { = 0} \hfill \\ f^{\prime}\left( \xi \right) \to A,\,\,\,f^{\prime \prime } \left( \xi \right) \to 0,\,\,\,\theta \left( \xi \right) \to 0\,,\,\,\Phi \left( \xi \right) \to 0,\,\,\,\,\,{\text{as}}\,\,\xi \to \infty . \hfill \\ \end{gathered} \right\} $$

Here

20 $$ \begin{aligned} \chi_{1} = & \left( {1 - \left( {\phi_{{Al_{2} O_{3} }} + \phi_{Cu} } \right)} \right)^{2.5} , \, \chi_{2} = \frac{1}{{\rho_{f} }}(\phi_{{Al_{2} O_{3} }} \rho_{{Al_{2} O_{3} }} + \phi_{Cu} \rho_{Cu} ) + \left( {1 - \phi_{hnf} } \right), \\ \chi_{3} = & \frac{1}{{\left( {\rho C_{p} } \right)_{f} }}\left( {\phi_{{Al_{2} O_{3} }} \left( {\rho C_{p} } \right)_{{Al_{2} O_{3} }} + \phi_{Cu} \left( {\rho C_{p} } \right)_{Cu} } \right) + \left( {1 - \phi_{hnf} } \right), \, \\ \chi_{4} = & \frac{{2k_{f} + \frac{\left( A \right)}{{\phi_{hnf} }} + 2\left( A \right) - 2k_{f} \phi_{hnf} }}{{2k_{f} + \frac{\left( A \right)}{{\phi_{hnf} }} + \left( A \right) - k_{f} \phi_{hnf} }},\,\,A = \phi_{{Al_{2} O_{3} }} k_{{Al_{2} O_{3} }} + \phi_{Cu} k_{Cu} \\ \,\chi_{5} = & \left( {1 - \left( {\phi_{{Al_{2} O_{3} }} + \phi_{Cu} } \right)} \right) \\ \end{aligned} $$

### Non-dimensional parameters

In the current research we have the following non-dimensional parameters obtained which is defined in the nomenclature table.

21 $$ \begin{gathered} A = \frac{a}{b},\,\,\Gamma = \frac{{\alpha_{1} b}}{{\mu_{f} }},\,\,\,K = \frac{{\upsilon_{f} }}{bk},\,\,\Pr = \frac{{\upsilon_{f} }}{{\alpha_{f} }},\,\,\alpha_{f} = \frac{{k_{f} }}{{\left( {\rho C_{p} } \right)_{f} }},Nr = \frac{{16\sigma^{*} T_{\infty }^{3} }}{{3k^{*} \upsilon_{f} \left( {\rho C_{p} } \right)_{f} }},\,\, \hfill \\ Ec = \frac{{U_{w}^{2} }}{{\left( {T_{w} - T_{\infty } } \right)\left( {C_{p} } \right)_{f} }},\,S = - V_{w} \sqrt {\frac{1}{{b\upsilon_{f} }}} ,\,\,Nt = \tau \frac{{D_{T} \left( {T_{w} - T_{\infty } } \right)}}{{\upsilon_{f} T_{\infty } }},\,\,Nb = \tau \frac{{D_{B} \left( {C_{w} - C_{\infty } } \right)}}{{\upsilon_{f} }},\,\, \hfill \\ E_{1} = \frac{Ea}{{kT_{\infty } }},\,\,K_{2} = \frac{{K_{1} r^{2} }}{b},\,\,\sigma_{1} = \frac{{\left( {T_{w} - T_{\infty } } \right)}}{{T_{\infty } }}. \hfill \\ \end{gathered} $$

## Engineering quantities

The Nusselt number ($Nu_{x}$) and Mass Transfer are written as

22 $$ Nu_{x} = \frac{{xq_{w} }}{{k_{ihnf} \left( {T_{w} - T_{\infty } } \right)}},Shu_{x} = \frac{{xj_{w} }}{{\left( {C_{w} - C_{\infty } } \right)}}. $$

Here $q_{w} ,j_{w}$ are the heat and mass flux terms. The simplified form are obtained as:

23 $$ Nu_{x} Re_{x}^{ - 0.5} = - \frac{{k_{thnf} }}{{k_{f} }}\theta^{\prime } \left( 0 \right),Shu_{x} Re_{x}^{ - 0.5} = - \Phi^{\prime } \left( 0 \right). $$

## Solution of the problem

In this analysis the two-dimensional laminar and steady flow of second grade hybrid nanofluid have been considered over the porous flat plate. In the flow profile we have considered the impact of thermal radiation on the second-grade fluid. Moreover, the impact of chemical reaction and activation energy are also analyzed on the concentration of the fluid. For calculating the solutions of the problem under consideration, we applied HAM method which is strong solutions and have many advantages and characteristics properties compared to other numerical methods. Therefore, keeping the above motivations in mind the present non-linear second grade fluid model of high order ODEs with physical boundary conditions have been solved numerically using HAM technique.

The HAM method is used due to the following characteristics properties.

- The accurate analytical solution has been obtained without linearization and discretization of the given differential equations.
- This is a strong method which handle both whether the problem is highly nonlinear or simple nonlinear problem.
- The convergency rate can be controlled of the obtained series solution by adjusting with the help of HAM.
- The HAM method can be found easy for computation and free from the errors related to rounding off.

Keeping these unique characteristics of the HAM method we prefer to use it for the analytical solutions. The initial guesses and linear operator for the solutions of the problem are defined as:

24 $$ \left[ \begin{gathered} f_{0} \left( \xi \right) = 1 + S - e^{ - \xi } , \hfill \\ \theta_{0} \left( \xi \right) = e^{ - \xi } , \hfill \\ \Phi_{0} \left( \xi \right) = e^{ - \xi } , \hfill \\ \end{gathered} \right] $$

The linear operator for the proposed second grade fluid problem can be expressed as:

25 $$ \left. \begin{gathered} L_{f} \left( \xi \right) = f^{\prime \prime \prime } (\xi ) - f^{\prime } \hfill \\ L_{\theta } (\xi ) = \theta^{\prime \prime } (\xi ) - \theta (\xi ), \hfill \\ L_{\Phi } (\xi ) = \Phi^{\prime \prime } (\xi ) - \Phi (\xi ), \hfill \\ \end{gathered} \right]. $$

The above equation for the solutions of the problem can be re-written as:

26 $$ \left[ \begin{gathered} L_{f} \left( {A_{1} + A_{2} {\text{e}}^{\xi } + A_{3} {\text{e}}^{ - \xi } } \right) = 0 \hfill \\ L_{\theta } \left( {A_{4} {\text{e}}^{\xi } + A_{5} {\text{e}}^{ - \xi } } \right) = 0 \hfill \\ L_{\Phi } \left( {A_{6} {\text{e}}^{\xi } + A_{7} {\text{e}}^{ - \xi } } \right) = 0. \hfill \\ \end{gathered} \right]. $$

where $A_{i} \left( {i = 1,2,3.....7} \right)$ are arbitrary constants.

### Zeroth order

This section explained the detail numerical scheme for HAM method. Therefore, starting from the first step which can be expressed as follows:

27 $$ (1 - \ell )L_{f} [f(\xi ,\ell ) - f_{0} (\xi )] = qh_{f} N_{f} [f(\xi ,\ell )], $$

28 $$ (1 - \ell )L_{\theta } [\theta (\xi ,\ell ) - \theta_{0} (\xi )] = qh_{\theta } N_{\theta } [f(\xi ,\ell ),\theta (\xi ,\ell )], $$

29 $$ (1 - \ell )L_{\Phi } [\Phi (\xi ,\ell ) - \Phi_{0} (\xi )] = qh_{\Phi } N_{\Phi } [f(\xi ,\ell )\,,\theta (\xi ,\ell ),\,\,\Phi (\xi ,\ell )], $$

here $$\xi$$ represents the embedding parameter which is used in during the HAM numerical scheme, and $$h_{f}$$$$h_{\theta }$$ and $$h_{\Phi }$$ are the embedding non-zero auxiliary parameters. $$N_{f}$$ , $$N_{\theta }$$ and $$N_{\Phi }$$ represents the nonlinear operators which can be defined as:

30 $$ \begin{aligned} N_{f} [f(\xi ,\ell )] = & \frac{{\partial^{3} f(\xi ,\ell )}}{{\partial \xi^{3} }} \\ & + \chi_{1} \chi_{2} \left[ \begin{gathered} \left( {f(\xi ,\ell )\frac{{\partial^{2} f(\xi ,\ell )}}{{\partial \xi^{2} }} - \left( {\frac{\partial f(\xi ,\ell )}{{\partial \xi }}} \right)^{2} } \right) + \hfill \\ \Gamma \left( \begin{gathered} 2\frac{\partial f(\xi ,\ell )}{{\partial \xi }}\frac{{\partial^{3} f(\xi ,\ell )}}{{\partial \xi^{3} }} \hfill \\ - \left( {\frac{{\partial^{2} f(\xi ,\ell )}}{{\partial \xi^{2} }}} \right)^{2} f(\xi ,\ell ) - f(\xi ,\ell )\frac{{\partial^{iv} f(\xi ,\ell )}}{{\partial \xi^{iv} }} \hfill \\ \end{gathered} \right) \hfill \\ \end{gathered} \right] - K\frac{\partial f(\xi ,\ell )}{{\partial \xi }} = 0 \\ \end{aligned} $$

31 $$ \begin{aligned} N_{\theta } [f(\xi ,\ell ),\theta (\xi ,\ell )] = & \chi_{4} \frac{{\partial^{2} \theta (\xi ,\ell )}}{{\partial \xi^{2} }}\left( {\chi_{4} + Nr} \right) \\ & + \chi_{3} \Pr \left( \begin{gathered} f(\ell ,\xi )\frac{\partial \theta (\xi ,\ell )}{{\partial \xi }} \hfill \\ + \frac{Ec}{{\chi_{1} }}\left[ {\left( {\frac{{\partial^{2} \theta (\xi ,\ell )}}{{\partial \xi^{2} }}} \right)^{2} + \Gamma \left( \begin{gathered} \frac{\partial \theta (\xi ,\ell )}{{\partial \xi }}\left( {\frac{{\partial^{2} \theta (\xi ,\ell )}}{{\partial \xi^{2} }}} \right)^{2} \hfill \\ - f(\xi ,\ell )\frac{{\partial^{2} \theta (\xi ,\ell )}}{{\partial \xi^{2} }}\frac{{\partial^{3} \theta (\xi ,\ell )}}{{\partial \xi^{3} }} \hfill \\ \end{gathered} \right)} \right] \hfill \\ \end{gathered} \right) = 0, \\ \end{aligned} $$

32 $$ \begin{aligned} N_{\Phi } [f(\xi ,\ell )\,,\theta (\xi ,\ell ),\,\,\Phi (\xi ,\ell )] = & \chi_{5} .\,\frac{{\partial^{2} \Phi (\xi ,\ell )}}{{\partial \xi^{2} }} + \frac{Nt}{{Nb}}\frac{{\partial^{2} \theta (\xi ,\ell )}}{{\partial \xi^{2} }} \\ &+ f(\xi ,\ell )\,\frac{\partial \Phi (\xi ,\ell )}{{\partial \xi }}.Sc - K_{2} .Sc.\Phi (\xi ,\ell )\,\left[ {\sigma_{1} \theta (\xi ,\ell ) + 1} \right]\exp \left[ { - \frac{{E_{1} }}{{\sigma_{1} \theta (\xi ,\ell )\, + 1}}} \right] = 0 \\ \end{aligned} $$

The initial condition to be satisfied are given as follows:

33 $$ f^{\prime}\left( {0,\ell } \right) = S,f^{\prime}(0,\ell ) = 1,\;f^{\prime } (\infty ,\ell ) = A\;and\;f^{\prime \prime } (\infty ,\ell ) = 0, $$

34 $$ \theta \left( {0,\ell } \right) = 1\;{\text{and}}\;\theta (\infty ,\ell ) = 0, $$

35 $$ \Phi \left( {0,\ell } \right) = 1\;{\text{and}}\;\Phi (\infty ,\ell ) = 0, $$

By putting the values of $\ell = 0$ then the Eqs. (27)–(29), can be expressed as follows:

36 $$ f(\xi ,0)\, = \,f_{0} (\xi ),\,\,\,\,\theta (\xi ,0)\, = \,\theta_{0} (\xi ),\,\,\,\,\Phi (\xi ,0) = \Phi_{0} (\xi ), $$

Similarly, by putting the values of $\ell = 1$ then the Eqs. (27)-(29), becomes:

37 $$ f(\xi ,1)\, = \,f(\,\xi ),\,\,\,\,\theta (\xi ,1)\, = \,\theta (\xi ),\,\,\,\,\Phi (\xi ,1) = \Phi (\xi ), $$

By employing the Taylor expansion in series form which is operated on the above equations. (36) and (37), we obtained the following results:

38 $$ f(\xi ,\ell ) = f_{0} (\xi ) + \sum\limits_{m = 1}^{\infty } {f_{m} (\xi )\ell^{m} ,} \;\;\;f_{m} (\xi ) = \frac{1}{m!}\frac{{\partial^{m} f(\xi ,\ell )}}{{\partial \xi^{m} }}|_{\ell = 0} , $$

39 $$ \theta (\xi ,\ell ) = \theta_{0} (\xi ) + \sum\limits_{m = 1}^{\infty } {\theta_{m} (\xi )\ell^{m} ,} \;\;\;\theta_{m} (\xi ) = \frac{1}{m!}\frac{{\partial^{m} \theta (\xi ,\ell )}}{{\partial \xi^{m} }}|_{\ell = 0} , $$

40 $$ \Phi (\xi ,\ell ) = \Phi_{0} (\xi ) + \sum\limits_{m = 1}^{\infty } {\phi_{m} (\xi )\ell^{m} ,} \;\;\;\;\Phi_{m} (\xi ) = \frac{1}{m!}\frac{{\partial^{m} \Phi (\xi ,\ell )}}{{\partial \xi^{m} }}|_{\ell = 0} , $$

In the above Eqs. (38)–(40), putting $\ell = 1$, the convergence of the series can be achieved in the following form:

41 $$ f(\xi ) = f_{0} (\xi ) + \sum\limits_{m = 1}^{\infty } {f_{m} (\xi ),} $$

42 $$ \theta (\xi ) = \theta_{0} (\xi ) + \sum\limits_{m = 1}^{\infty } {\theta_{m} (\xi ),} $$

43 $$ \Phi (\xi ) = \Phi_{0} (\xi ) + \sum\limits_{m = 1}^{\infty } {\Phi_{m} (\xi ),} $$

### The mth order

The *m*th order form of the present proposed problem for the HAM numerical scheme can be written as follows:

44 $$ L_{f} [f_{m} (\xi ) - \eta_{m} f_{m - 1} (\xi )] = h_{f} R_{m}^{f} m(\xi ), $$

45 $$ L_{\theta } [\theta_{m} (\xi ) - \eta_{m} \theta_{m - 1} (\xi )] = h_{\theta } R_{m}^{\theta } (\xi ), $$

46 $$ L_{\Phi } [\Phi_{m} (\xi ) - \eta_{m} \Phi_{m - 1} (\xi )] = h_{\Phi } R_{m}^{\Phi } m(\xi ), $$

47 $$ f_{m} (0) = 0,\,\,\,\,{\text{and}}\,\,\,\,f_{m} (\infty ) = 0, $$

48 $$ \theta_{m} (0) = 0,\,\,\,{\text{and}}\,\,\,\theta_{m} (\infty ) = 0, $$

49 $$ \phi_{m} (0) = 0,\,\,\,\,\,\,{\text{and}}\,\,\,\,\phi_{m} (\infty ) = 0, $$

In the above Eqs. (44)–(46), the terms $R_{m}^{f} m(\xi )$ , $R_{m}^{\theta } m(\xi )$ and $R_{m}^{\Phi } m\left( \xi \right)$ are defined as follows:

50 $$ R_{m}^{f} m(\xi ) = f_{m - 1}^{\prime \prime \prime } + \chi_{1} \chi_{2} \left[ \begin{gathered} \left( {\sum\limits_{k = 0}^{m - 1} {f_{m - 1 - k} } f_{k}^{\prime \prime } - \sum\limits_{k = 0}^{m - 1} {f_{m - 1 - k}^{^{\prime}} } f_{k}^{^{\prime}} } \right) + \hfill \\ \Gamma \left( \begin{gathered} 2\sum\limits_{k = 0}^{m - 1} {f_{m - 1 - k}^{\prime } } f_{k}^{\prime \prime \prime } - \sum\limits_{k = 0}^{m} {\left( {\sum\limits_{1 = 0}^{k} {f^{\prime \prime } } f_{k - 1}^{\prime \prime } } \right)} f_{m - k} \hfill \\ - \sum\limits_{k = 0}^{m - 1} {f_{m - 1 - k} } f_{k}^{\prime \prime \prime \prime } \hfill \\ \end{gathered} \right) \hfill \\ \end{gathered} \right] - Kf_{m - 1}^{\prime \prime \prime } = 0, $$

51 $$ R_{m}^{\theta } m(\xi ) = \chi_{4} \theta_{m - 1}^{\prime \prime } \left( {\chi_{4} + Nr} \right) + \chi_{3} \Pr \left( \begin{gathered} \sum\limits_{k = 0}^{m - 1} {f_{m - 1 - k} } \theta_{k}^{\prime } \hfill \\ + \frac{Ec}{{\chi_{1} }}\left[ \begin{gathered} \sum\limits_{k = 0}^{m - 1} {f_{m - 1 - k}^{\prime \prime } } f_{k}^{^{\prime\prime}} \hfill \\ + \Gamma \left( \begin{gathered} \sum\limits_{k = 0}^{m} {\left( {\sum\limits_{1 = 0}^{k} {f^{\prime } } f_{k - 1}^{\prime \prime } } \right)} f_{m - k}^{\prime \prime } \hfill \\ - \sum\limits_{k = 0}^{m} {\left( {\sum\limits_{1 = 0}^{k} f f_{k - 1}^{\prime \prime } } \right)} f_{m - k}^{\prime \prime \prime } \hfill \\ \end{gathered} \right) \hfill \\ \end{gathered} \right] \hfill \\ \end{gathered} \right) = 0, $$

52 $$ \begin{aligned} R_{m}^{\Phi } m(\xi ) = & \chi_{5} .\,\Phi_{m - 1}^{\prime \prime } + \frac{Nt}{{Nb}}\theta_{m - 1}^{\prime \prime } + \sum\limits_{k = 0}^{m - 1} {f_{m - 1 - k} } \Phi_{k}^{\prime } .Sc \\ & - K_{2} .Sc\left[ {\sigma_{1} \sum\limits_{k = 0}^{m - 1} {\Phi_{m - 1 - k} } \theta_{k} + \Phi_{m - 1} } \right]\exp \left[ { - \frac{{E_{1} }}{{\sigma_{1} \theta_{m - 1} + 1}}} \right] = 0. \\ \end{aligned} $$

53 $$ \eta_{m} = \left\{ {\begin{array}{*{20}c} {0, m \le 1} \\ {1, m > 1.} \\ \end{array} } \right. $$

The final general solution of the current proposed mode is obtained by using the particular solutions:

54 $$ f_{m} (\xi ) = f_{m}^{*} (\xi ) + A_{1} + A_{2} {\text{e}}^{\xi } + A_{3} {\text{e}}^{ - \xi } , $$

55 $$ \theta_{m} (\xi ) = \theta_{m}^{*} (\xi ) + A_{4} {\text{e}}^{\xi } + A_{5} {\text{e}}^{ - \xi } , $$

56 $$ \Phi_{m} (\xi ) = \Phi_{m}^{*} (\xi ) + A_{6} {\text{e}}^{\xi } + A_{7} {\text{e}}^{ - \xi } . $$

Which are the final general solutions of the present proposed model by using the HAM numerical scheme.

## Results and discussion

**Figure 2:**
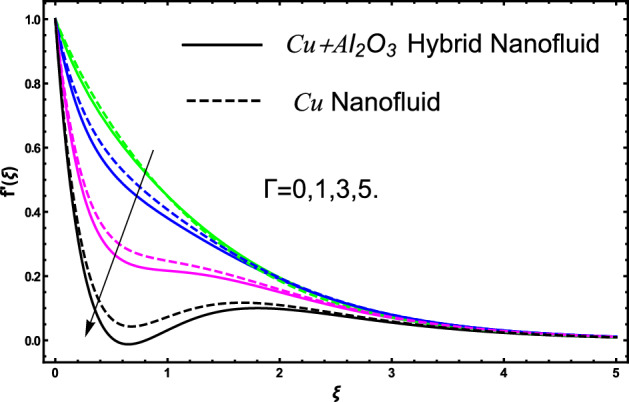
The fluctuation in velocity $f^{^{\prime}} \left( \xi \right)$ profile against escalating values of $\Gamma$.

**Figure 3:**
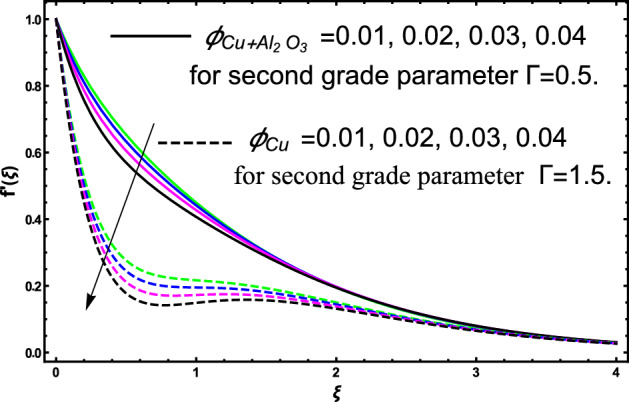
The fluctuation in velocity $f^{^{\prime}} \left( \xi \right)$ profile against various values of $\phi_{{Al_{2} O_{3} + Cu}}$ and $\phi_{Cu}$ for elevated values of $\Gamma$.

**Figure 4:**
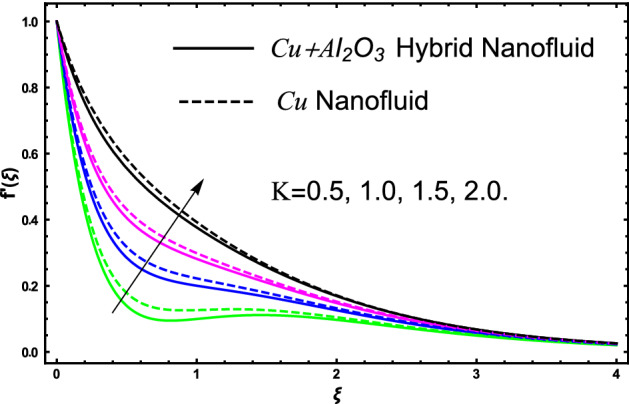
The fluctuation produces in the velocity $f^{^{\prime}} \left( \xi \right)$ profile against escalating values of $K$.

**Figure 5:**
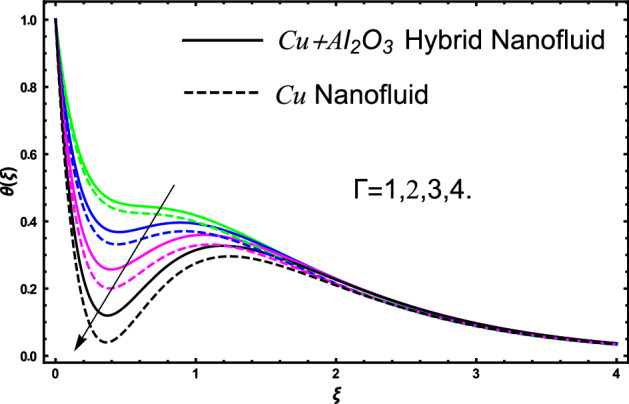
The fluctuation in temperature $\theta \left( \xi \right)$ profile of the fluid against elevated values of $\Gamma$.

**Figure 6:**
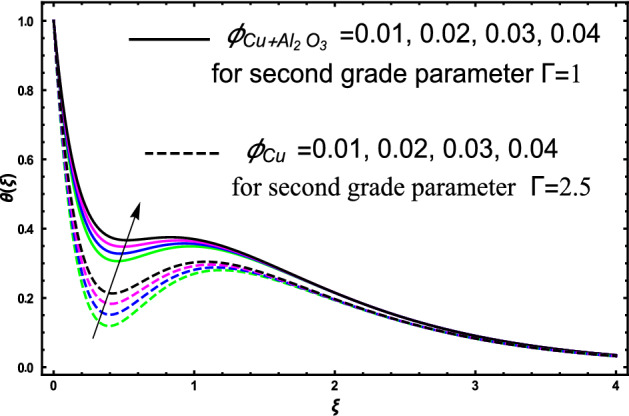
The fluctuation in temperature $\theta \left( \xi \right)$ profile against various values of $\phi_{{Al_{2} O_{3} + Cu}}$ and $\phi_{Cu}$ for different values of $\Gamma$.

**Figure 7:**
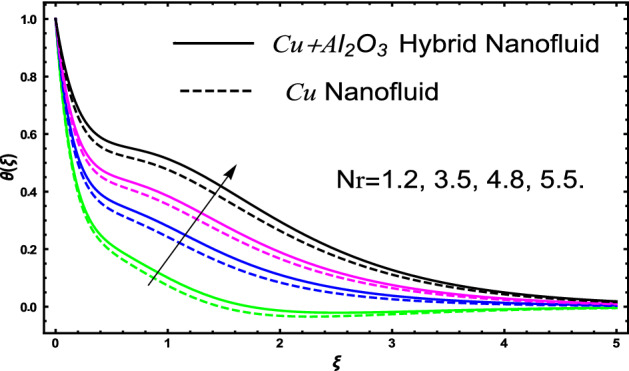
The fluctuation in temperature $\theta \left( \xi \right)$ profile versus accelerated values of $Nr$.

**Figure 8:**
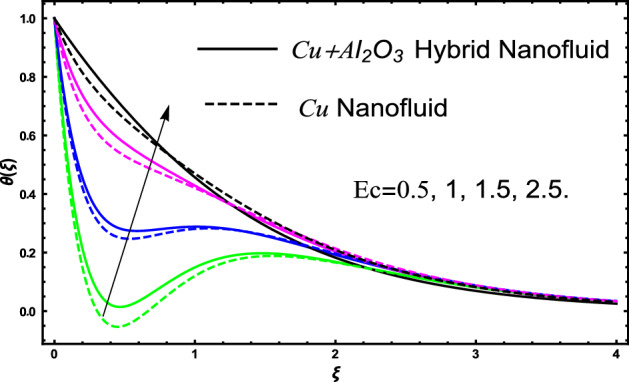
The fluctuation occur in temperature $\theta \left( \xi \right)$ versus large values of $Ec$.

**Figure 9:**
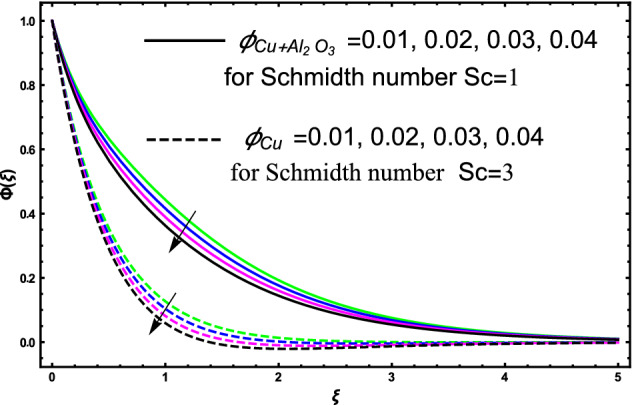
The fluctuation of concertation $\phi \left( \xi \right)$ against elevated values of $\phi_{{Al_{2} O_{3} + Cu}}$ and $\phi_{Cu}$ for increasing values of $Sc$.

**Figure 10:**
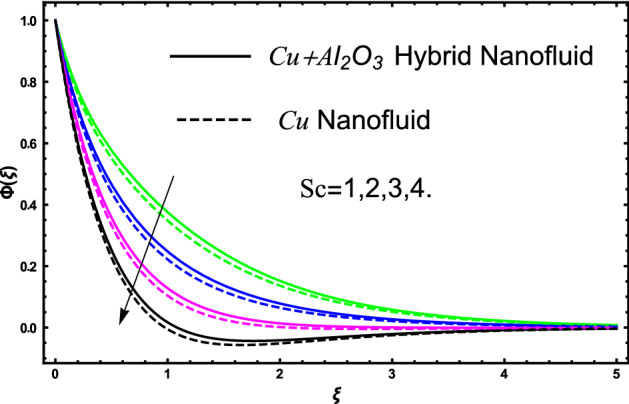
The fluctuation of concertation $\phi \left( \xi \right)$ versus greater values of $Sc$.

**Figure 11:**
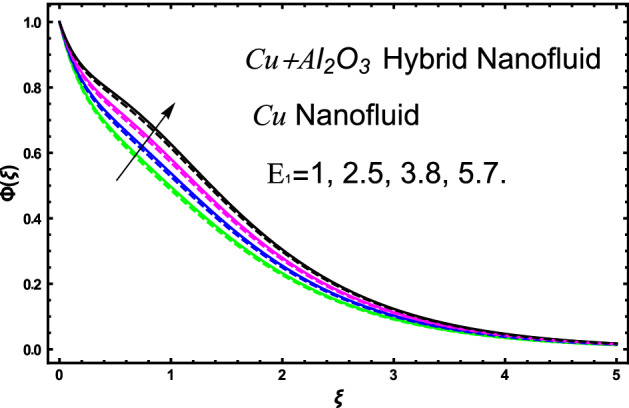
The fluctuation of concertation $\phi \left( \xi \right)$ versus escalating values of $E_{1}$.

**Figure 12:**
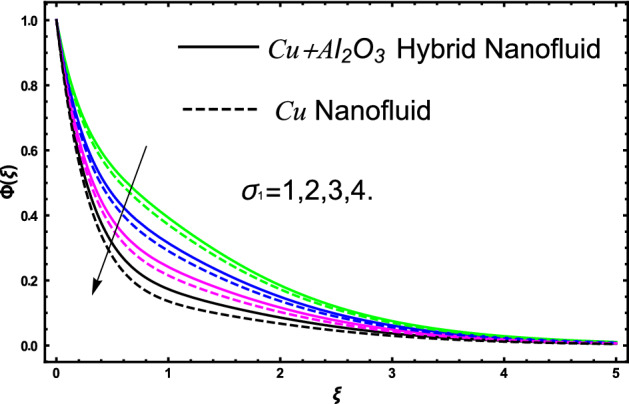
The fluctuation of concertation $\phi \left( \xi \right)$ versus extended values of $\sigma_{1}$.

**Figure 13:**
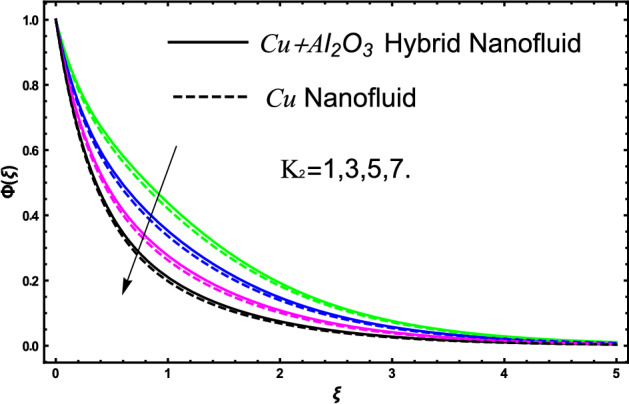
The fluctuation of concertation $\phi \left( \xi \right)$ versus escalating values of $K_{2}$.

The current research inspects the incompressible, laminar and steady flow of second grade $Al_{2} O_{3} - Cu$/engine oil based hybrid nanofluid passing over a horizontal porous flat plate. The presence of radiation, chemical reaction, activation energy, Brownian motion and thermophoresis are highlighted in current work. The proposed model has system of higher-order PDE’s which is transformed to the non-linear system of ODE’s. For the solution methodology HAM numerical scheme is applied. Keeping in mind the applications of hybrid nanofluid this research carried out a detail analysis by taking engine oil as base fluid and two nanoparticles $Al_{2} O_{3}$ and $Cu$ are added for the advance cooling applications in engineering problems. From many early studies it was found that hybrid nanofluid is more effective compared to simple mon0 nano liquids and regular fluids. Therefore, hybrid nanofluid is considered in the current research. In addition to this all the physical parameters are portrayed through graphs to highlight the behavior of each parameter in the dynamics of the fluid. The impact of volume fraction on the flow, heat and mass are also evaluated and presented in graphs. Moreover, the comparative analysis between hybrid and nanofluid is carried out and found that hybrid nanofluid performed well as compared to nanofluid and regular fluid. The engineering quantities obtained from the present research have been presented in tables. Moreover, the geometry of flow is given in Fig. 1. The influence of $\Gamma$, $\phi_{{Al_{2} O_{3} + Cu}}$,$\phi_{Cu}$,$K$ on the velocity profile is portrayed in Figs. (2, 3, 4) respectively. Similarly, thee impact of $\Gamma$, $\phi_{{Al_{2} O_{3} + Cu}}$,$\phi_{Cu}$,$Nr$ and $Ec$ on the temperature profile are plotted in Figs. (5, 6, 7, 8) respectively. The effect of $\phi_{{Al_{2} O_{3} + Cu}}$,$\phi_{Cu}$,$Sc$,$E_{1}$,$\sigma_{1}$ and $K_{2}$ are highlighted on the concentration of the second grade fluid in Figs. (9, 10, 11, 12, 13) respectively. Furthermore, from Figs. (2, 3, 4, 5, 6, 7, 8, 9, 10, 11, 12, 13) all the graphs show the comparison between mixture of $Al_{2} O_{3} - Cu$/engine oil based hybrid nanofluid with single nanoparticle $Cu$/engine oil based nanofluid. From the comparison it is depicted that the mixture $Al_{2} O_{3} - Cu$/engine oil-based hybrid nanofluid have great thermal performance as compared to $Cu$/engine oil based unitary nanofluid.

In all the plots for energy, mass and momentum we have compared our obtained solutions for $Al_{2} O_{3} - Cu$/engine oil hybrid nano liquids with the unitary fluid containing $Cu$ nanoparticles. From the compared results we see that thermal performance of engine oil was effective by adding $Al_{2} O_{3} - Cu$ nanoparticles. The impact of second grade fluid parameter $\Gamma$ on flow profile is portrayed in Fig. 2. From the figure the fluctuation in velocity against various values of $\Gamma$ it can be depicted that higher the values of $\Gamma$ the velocity of fluid get slow down. This is true because for increasing the $\Gamma$ it produces instability in fluid and due this produces significant increment in momentum boundary layer, as result fluid velocity declines. Furthermore, it can also be inspected form the figure that for $\Gamma = 0$ shows the simple viscous fluid and it can be compared with the second-grade fluid. From the comparative analysis it elucidates that second-grade fluid have higher density as compared to viscous fluid. The flow of second grade hybrid nanofluid for escalating values of volume fraction $\phi_{{Al_{2} O_{3} + Cu}}$ and $\phi_{Cu}$ are portrayed in Fig. 3. Moreover, in the figure we have plotted the impact of volume fraction hybrid nanofluid when $\Gamma = 0.5$, and the impact of nanofluid volume friction when $\Gamma = 1.5$. The fluctuation produces in velocity of the fluid against escalating values of volume friction can improve the concertation within the fluid due to which the viscous forces become dominant and consequently the fluid velocity decreases and slowdown. Furthermore, in the figure we developed the comparative analysis between the mixture of nanoparticles and a single nanoparticle in engine and found that the performance of fluid having two types of particles is more effective as compared to fluid containing one type of nanoparticles. Figure 4 visualizes the impact of porous medium on the velocity profile of the second-grade fluid. From the figure it can be clearly seen that an increase in the porosity parameter an increasing behavior in the fluid velocity is noticed. This fluctuation in the velocity is occur due to the fact that enlarge the pores in the surface causes a reduction in the resistive forces between the fluid and surface consequently a smooth flow occurs due to which velocity of the second-grade fluid get higher. A comparison is shown in figure for engine oil have $Al_{2} O_{3} - Cu$ and only $Cu$ from the comparison it was clearly noticed that engine performance is appreciated by considering $Al_{2} O_{3} - Cu$ mixture in engine oil.

Figure 5 investigates the trends of second grade parameter $\Gamma$ on the temperature of the fluid. From the figure it can be inspected that the temperature of the fluid gets lowered for the increment of $\Gamma$ it is due to the fact that second grade parameter $\Gamma$ increase the density of the fluid. Therefore, higher the density will decrease reduced the temperature it is because density is inversely proportional to the temperature of the fluid. Therefore, increasing $\Gamma$ will increase the density consequently the fluid temperature declines. The temperature of hybrid nanofluid for different values of volume fraction $\phi_{{Al_{2} O_{3} + Cu}}$ and $\phi_{Cu}$ are highlighted in Fig. 6. Moreover, in the figure we have plotted the impact of volume fraction hybrid nanofluid when $\Gamma = 1.5$, and the impact of nanofluid volume friction when $\Gamma = 2.5$. The thermal profile shows that elevated values of the nanoparticles concentration will increases the friction between the particles in the fluid which make an increase in the temperature of the working fluid. Secondly, the increase in the temperature in this case is since higher the concentration a greater collision will occur within the fluid as result kinetic energy increase which responsible for the boost up the temperature. Furthermore, in the figure we developed the comparative analysis between the mixture of nanoparticles and a single nanoparticle in engine and found that the performance of hybrid nanofluid have good thermal properties. Figure 7 highlighted the impact of $Nr$ on the fluid temperature. The fluctuation in temperature is elucidated and found that elevated values of $Nr$ will increase the temperature of the fluid in both the cases nanofluid and hybrid nanofluid. The radiation causes an increase in the total internal energy of the fluid due to which temperature of the fluid get higher. The fluctuation in temperature of the fluid versus elevated values of $Ec$ is highlighted in Fig. 8. This variation in temperature profile is occur because higher the $Ec$ temperature profile attains high values. This trends in the temperature are since higher values of $Ec$ improve the temperature profile because there is maximum friction within the fluid due to which temperature get higher. Higher $Ec$ means a maximum heat transport characteristics appears in the fluid due to maximum resistive forces within the fluid and hence temperature get higher.

The concentration of hybrid nanofluid for different values of volume fraction $\phi_{{Al_{2} O_{3} + Cu}}$ and $\phi_{Cu}$ are displayed in Fig. 9. In the present figure at the same time, we show the variation in concentration profile for two different values of $Sc = 1$ and $Sc = 3$. It is worth noting from the figure that the impact of volume fraction of hybrid nanofluid $\phi_{{Al_{2} O_{3} + Cu}}$ is plotted for $Sc = 1$ and in case of nanofluid volume fraction $\phi_{Cu}$ the Schmidt number $Sc = 3$. The concertation gets lower as we gradually increase nanoparticles volume fraction in both the cases hybrid nanofluid and nanofluid as well. Figure 10 display the evolvement of Schmidt $Sc$ on the concertation of the fluid. From the figure the higher the values of $Sc$ a decline have been noticed in the concertation of the fluid in both the cases hybrid nanofluid and nanofluid. It is worth noting that $Sc$ is the ratio among the kinematic viscosity to the molecular diffusion coefficient. When the $Sc$ taken higher as a result the viscosity become dominant consequently the concertation profile decreases as shown in the figure. The non-dimensional parameter of activation energy $E_{1}$ are highlighted in Fig. 11. From the figure by selecting the high numerical values of $E_{1}$ increases the boundary layer thickness of the concertation profile consequently an increase is noticed in the concertation of the fluid flow due to which mass concentration get higher for hybrid nanofluid and unitary nanofluid as shown in the figure. Figure 12 elucidates the impact of temperature difference parameter $\sigma_{1}$ on the concertation profile of the fluid. An increase in the non-dimensional parameter $\sigma_{1}$ causes concertation reduction because $\sigma_{1}$ reduces the concentration within the fluid due to which a decrement is noticed in both hybrid nanofluid and mono nanofluid as shown in the figure. Figure 13 elucidates the impact of chemical reaction parameter $K_{2}$ on the concertation profile of the fluid. An increase in the non-dimensional parameter $K_{2}$ is responsible for the thickening of the mass transfer in the boundary layer region due to which the concentration reduces in both the cases hybrid nanofluid and unitary nanofluid as shown in figure.

Table 1 shows the thermos-physical properties of engine oil base fluid and aluminum oxide $\left( {Al_{2} O_{3} } \right)$ and Copper $\left( {Cu} \right)$ nanoparticles. Table 2 presents the numerical values of the skin friction versus various parameters. From the table the impact of $\phi$, $\Gamma$, $Ec$,$Nu_{x} \,{\text{for}}\,Cu$ and $Nu_{x} \,\,{\text{for}}\,\,Al_{2} O_{3} + Cu$ on the Nusselt number. The Nusselt number elevated for higher values of $\phi$ and $Ec$ while the skin friction get lower in the case of second grade fluid parameter Similarly, Table 3 presented the calculation of Sherwood number (Sh) for various flow parameters. From the table Sh get lower for higher values of $E_{1}$, $Sc$ and $K_{2}$ for hybrid nanofluid and mono nanofluid. Table 4 elaborates the relative comparison for skin friction and Nusselt using nanoparticles volume fraction, magnetic effect and Prandtl number versus published study. It can be clearly observed that both results are in best settlement with each other and ensure the accuracy of the present outcomes.

**Table 1:** Thermal properties of base fluid (EO), and nano-meter sized particles (*Cu)* and *(Al*_*2*_*O*_*3*_) are given in refs.^11, 61^.

Material	$\rho (Kgm^{ - 3} )$	$c_{p} (JKg^{ - 1} K^{ - 1} )$	$k(wm^{ - 1} K^{ - 1} )$	$\beta \times 10^{ - 5} (K^{ - 1} )$	$\sigma$ $\left( {\Omega .m} \right)^{ - 1}$
EO	884	1910	0.144	70	1.57*10^–8^
*Al*_*2*_*O*_*3*_	3970	765	40	0.85	10^–10^
*Cu*	8933	385	400	1.67	10^–7^

**Table 2:** $Nu_{x} Re_{x}^{ - 0.5}$ numerical values for versus various parameters.

$\phi$	$\Gamma$	$Ec$	$$\begin{gathered} Nu_{x} \hfill \\ Cu \hfill \\ \end{gathered}$$	$$\begin{gathered} Nu_{x} \hfill \\ Al_{2} O_{3} + Cu \hfill \\ \end{gathered}$$
0.01	1	1	1.287653120234	1.3019267387653
0.02			1.3032901282132	1.3676281080329
0.03			1.3787343416221	1.40129348478734
	1.2		1.0023876215232	0.8986002387621
	1.3		0.7320183456215	0.5219867320183
		3	1.7432100654713	1.9432198774321
		5	2.329872082320	2.89732162876531

**Table 3:** $Shu_{x} Re_{x}^{ - 0.5}$ numerical values versus various parameters.

$Sc$	$E_{1}$	$K_{2}$	$$\begin{gathered} Shu_{x} \hfill \\ Cu \hfill \\ \end{gathered}$$	$$\begin{gathered} Shu_{x} \hfill \\ Al_{2} O_{3} + Cu \hfill \\ \end{gathered}$$
0.3	0.01	0.4	1.432102876531	1.430432982102
0.5			1.375439803290	1.3721879654398
0.7			1.323876787343	1.321098433876787
	0.02		1.3287002387654	1.32643201870023
	0.03		1.295437320183	1.2921908764543732
		0.6	1.3265743210065	1.32421096574321
		0.8	1.1303298720825	1.4303276421028

**Table 4:** Relative comparison of the present work with the published literature. While keeping $E_{1} = \sigma_{1} = \Gamma = 0.$

Parameters				Sreedevi et al.^46^	Present Work	Sreedevi et al.^46^	Present Work
$\phi_{1}$	$\phi_{2}$	*M*	*Pr*	$f^{\prime\prime}\left( 0 \right)$	$f^{\prime\prime}\left( 0 \right)$	$- \theta^{\prime}\left( 0 \right)$	$- \theta^{\prime}\left( 0 \right)$
0.01				0.14572	0.145813	1.63360	1.633711
0.02				0.10529	0.105431	1.50246	1.502522
0.03	0.01			0.07013	0.070234	1.36258	1.362614
	0.02			0.14572	0.145817	1.63434	1.634444
	0.03			0.11054	0.110624	1.38204	1.382212
		0.1		0.08104	0.081212	1.16310	1.163314
		0.5		0.10522	0.105324	1.16288	1.162976
		1.0		0.05120	0.051316	1.13220	1.132393
			6.2	0.02440	0.024534	1.11348	1.113586
			5.2	0.10522	0.105329	0.92175	0.921712
			4.2	0.10545	0.105611	1.04739	1.047425

## Conclusion

This section provides the impact of heat and mass transfer of incompressible second grade engine oil base hybrid nanofluid flow over a porous horizontal stretching porous flat plate. Furthermore, a comparison between $Al_{2} O_{3} - Cu$/engine oil based hybrid nanofluid with single nanoparticle $Cu$/engine oil based nanofluid is carried out and found that the thermal efficiency of engine oil is enhance in case of $Al_{2} O_{3} - Cu$/engine oil based hybrid nanofluid. Moreover, this study addresses the impact of thermal radiation, chemical reaction, and activation energy. For the solutions of high non-linear ordinary differential equations, a strong numerical technique HAM is used. For plotting the graphs, a computational software MATHEMATICA is used. All the flow parameters are highlighted through figures and discussed in detail their physical aspect on the flow heat and mass transfer. During the present analysis we get the following outcomes:

- The second-grade fluid parameter $\Gamma$ reduces the flow and heat profiles.
- The higher values of volume fraction of hybrid nanofluid and mono nanofluid reduces the velocity of the fluid flow.
- The higher values of volume fraction of hybrid nanofluid and mono nanofluid reduces the fluid temperature.
- A comparison between $Al_{2} O_{3} - Cu$/engine oil-based hybrid nanofluid and $Cu$/engine oil based nanofluid is carried out and found that hybrid nanofluid is more effective as compared to nanofluid.
- Increasing the chemical reaction $K_{2}$ reduces the concentration profile.
- Increasing the activation energy $E_{1}$ increases the concertation of the fluid.
- The Nusselt number get higher for higher values of $\phi$ and $Ec$ while the skin friction get lower in the case of second grade fluid parameter in both the cases hybrid nanofluid and mono nanofluid.
- Sherwood number get lower for higher values of $E_{1}$, $Sc$ and $K_{2}$ for hybrid nanofluid and mono nanofluid.

## Data Availability

All data used in this manuscript have been presented within the manuscript. No data is hidden or restricted.

## List of symbols

$$C_{w}$$: Surface concentration
$$C_{\infty }$$: Ambient concertation
$$T_{w}$$: Surface temperature
$$T_{\infty }$$: Ambient temperature
$$(u,v)$$: Velocity components
$$q_{r}$$: Thermal radiation
$U$: Free stream velocity
$\alpha_{1}$: Second grade fluid parameter
$$k$$: Porosity parameter
$$D_{B}$$: Brownian diffusion
$$D_{T}$$: Thermophoretic diffusion
$$K_{1} r^{2}$$: Chemical reaction
$$E_{a}$$: Activation energy
$\rho_{hnf}$: Density of hybrid nanofluid
$\mu_{hnf}$: Kinematic viscosity of hybrid nanofluid
$k_{hnf}$: Thermal conductivity of hybrid nanofluid
$C_{p}$: Specific heat capacitance,
$\left( {D_{B} } \right)_{hnf}$: Mass diffusivity of hybrid nanofluid.
$$q_{w}$$: Heat flux
$$j_{w}$$: Mass flux,
$${\text{Re}}$$: Reynold number
$$Nu_{x}$$: Nusselt number
$k_{r}^{2} \left( {\frac{T}{{T_{\infty } }}} \right)^{n} \exp \left( { - \frac{{E_{a} }}{k*T}} \right)$: Revised Arrhenius equation
$\frac{\partial }{\partial t}$: Time dependent derivative
$V$: Velocity filed vector form
${\varvec{T}}$: Cauchy stress tensor
$b$: Extending rate of the stretching porous sheet
$$p$$: Pressure
$$\alpha_{1} ,\,\,\alpha_{2}$$: Material variables
$${\varvec{A}}_{m1} \,{\text{and}}\,{\varvec{A}}_{m2}$$: Kinematic tensors
$$I$$: Identity tensor
$$A$$: Stretching parameter
$\Gamma$: Second grade fluid parameter
$$K$$: Porosity parameter
$$\Pr$$: Prandtl number
$$\alpha_{f}$$: Thermal diffusivity
$\sigma_{1}$: Temperature difference parameter
$$Nr$$: Radiation parameter
$$Ec$$: Eckert number
$S$: Suction/injection parameter
$$Nt$$: Thermophoresis parameter
$$Nb$$: Brownian parameter
$E_{1}$: Activation energy constant
$K_{2}$: Chemical reaction parameter
$$Shu_{x}$$: Mass transfer
